# Antiretroviral Therapy and Mortality in Rural South Africa: A Comparison of Causal Modeling Approaches

**DOI:** 10.1093/aje/kwy065

**Published:** 2018-03-23

**Authors:** Catherine E Oldenburg, George R Seage, Frank Tanser, Victor De Gruttola, Kenneth H Mayer, Matthew J Mimiaga, Jacob Bor, Till Bärnighausen

**Affiliations:** 1 Department of Epidemiology, Harvard T.H. Chan School of Public Health, Boston, Massachusetts; 2 Francis I. Proctor Foundation, University of California, San Francisco, San Francisco, California; 3 Africa Health Research Institute, Durban and Somkhele, South Africa; 4 Department of Epidemiology, Faculty of Health Sciences, University of KwaZulu-Natal, Durban, South Africa; 5 Department of Biostatistics, Harvard T.H. Chan School of Public Health, Boston, Massachusetts; 6 The Fenway Institute, Fenway Community Health, Boston, Massachusetts; 7 Department of Global Health and Population, Harvard T.H. Chan School of Public Health, Boston, Massachusetts; 8 Department of Medicine, Beth Israel Deaconess Medical Center, Boston, Massachusetts; 9 Department of Behavioral and Social Sciences and Department of Epidemiology, Institute for Community Health Promotion, School of Public Health, Brown University, Providence, Rhode Island; 10 Department of Global Health, School of Public Health, Boston University, Boston, Massachusetts; 11 Research Department of Infection and Population Health, Centre for Sexual Health, University College London, London, United Kingdom; 12 Heidelberg Institute of Public Health, University of Heidelberg, Heidelberg, Germany

**Keywords:** antiretroviral therapy, causal inference, HIV, marginal structural models, mortality, regression discontinuity, South Africa

## Abstract

Estimation of causal effects from observational data is a primary goal of epidemiology. The use of multiple methods with different assumptions relating to exchangeability improves causal inference by demonstrating robustness across assumptions. We estimated the effect of antiretroviral therapy (ART) on mortality in rural KwaZulu-Natal, South Africa, from 2007 to 2011, using 2 methods with substantially different assumptions: the regression discontinuity design (RDD) and inverse-probability–weighted (IPW) marginal structural models (MSMs). The RDD analysis took advantage of a CD4-cell-count–based threshold for ART initiation (200 cells/μL). The 2 methods yielded consistent but nonidentical results for the effect of immediate initiation of ART (RDD intention-to-treat hazard ratio (HR) = 0.66, 95% confidence interval (CI): 0.35, 1.26; RDD complier average causal effect HR = 0.56, 95% CI: 0.41, 0.77; IPW MSM HR = 0.49, 95% CI: 0.42, 0.58). Although RDD and IPW MSM estimates have distinct identifying assumptions, strengths, and limitations in terms of internal and external validity, results in this application were similar. The differences in modeling approaches and the external validity of each method may explain the minor differences in effect estimates. The overall consistency of the results lends support for causal inference about the effect of ART on mortality from these data.

The most commonly used epidemiologic methods for identification of causal effects rely on the untestable assumption of no unmeasured confounding to achieve exchangeability (i.e., that the counterfactual risk of outcome for each exposure status is the same in the exposed and the unexposed) (1, 2). Recently, nonrandomized study designs have been classified into 2 broad categories based primarily on the underlying assumptions for internal validity (3). “Quasi-experimental” study designs have been defined as those that utilize an exogenous source in variation in exposure assignment (e.g., an instrumental variable). Because the source of variation in treatment assignment is not related to the causal structure, these designs do not require the typical assumption of no unmeasured confounding to achieve exchangeability, and they do not require explicit modeling of or adjustment for covariates (4). However, in order for valid causal inferences to be drawn, these designs each require their own set of assumptions to be met, some of which are not empirically verifiable (5).

The second category, “nonexperimental” studies, encompasses study designs in which exposure allocation is modeled as a process that is endogenous to the causal structure under study (3). Because exposure is allocated nonrandomly, these study designs require measurement of and adjustment for all potential confounders. The models used under this umbrella must explicitly model all potential confounders to identify causal effects, and thus they require the assumption of no unmeasured or residual confounding.

In addition to differences in assumptions for exchangeability, the external validity of results differs by causal inference method. To achieve exchangeability, restricting the analytical population of a quasi-experimental study to persons with values that are close to the exogenous source of exposure allocation is often necessary. Without untestable assumptions, these results will only be generalizable to target populations with a similar distribution of values. Conversely, nonexperiments may be generalizable to a larger population, as they estimate the association in the entire study population. The answer to the same research question can thus differ depending on whether it has been derived from a quasi-experiment or a nonexperiment.

In controlled trials of antiretroviral therapy (ART) among persons with human immunodeficiency virus (HIV) infection, immediate initiation of ART has been shown to reduce mortality in comparison with delayed initiation (6–8). Real-world causal effects outside of tightly controlled randomized trials may differ because of differences in adherence (e.g., without the counseling provided in a trial) or drug stock-outs. However, identification of the effect of ART on mortality in observational data using traditional covariate-adjustment regression methods is limited by confounding by indication. ART initiation is a function of cluster of differentiation 4 (CD4)-positive T-cell count, which is also an indicator of disease progression and thus is associated with mortality. Failing to account for CD4 cell count will therefore lead to incorrect inferences (9). However, standard covariate adjustment cannot be used if the exposure is time-varying, because time-varying confounders are affected by previous treatment. For time-fixed exposures, standard regression could be used, although it is not ideal because the hazard ratio is noncollapsible. Here we compare the assumptions and external validity of the regression discontinuity design (RDD) (10–14), classified as a quasi-experimental approach, with that of inverse- probability–weighted (IPW) marginal structural models (MSMs) (9), classified as a nonexperimental approach. We then illustrate the comparison of approaches with an application to assessment of the causal effect of ART on survival in rural KwaZulu-Natal, South Africa (Table 1).

**Table 1.** Comparison of Regression Discontinuity Design and Inverse-Probability–Weighted Marginal Structural Models as Causal Modeling Strategies for Initiation of Antiretroviral Therapy Among Persons With Human Immunodeficiency Virus Infection

Element of Study Design	Regression Discontinuity Design	Inverse-Probability–Weighted Marginal Structural Model
Causal question	The causal effect of immediate ART eligibility on mortality	The causal effect of ART initiation at entry to care on mortality
Effect estimated	Local treatment effect	Average causal effect
Key assumption	Potential outcomes are continuous at the threshold (continuity assumption)	No unmeasured confounding
Handling of confounding	Whether or not a person near the threshold presents to the clinic just above or just below the threshold is assumed to be random	Creating a pseudopopulation where treatment is random conditional on measured covariates
Statistical power relative to standard cohort methods	Strongly reduced	Reduced

Abbreviation: ART, antiretroviral therapy.

## METHODS

### Regression discontinuity design

The RDD can be used in settings where an exposure is assigned by a threshold rule based on a continuously measured variable (the assignment variable) (10, 15–19). Persons who present for care close to the threshold value who are measured just above and just below the threshold are expected to be similar with respect to the distributions of measured and unmeasured covariates. Patients immediately above and below the threshold value are expected to be exchangeable (see Web Appendix 1, available at https://academic.oup.com/aje).

A discontinuity in treatment assignment occurs when the probability of receiving treatment given that a patient is above the threshold does not equal the probability of receiving treatment given that the patient is below the threshold. Causal effects can then be estimated in a small neighborhood around the threshold. This process has been formally described elsewhere (10). When the assignment procedure is deterministic (when the threshold rule perfectly determines treatment), the procedure is known as “sharp regression discontinuity.” In the probabilistic case, where not all patients receive the treatment they are assigned by the threshold, the procedure is known as “fuzzy regression discontinuity.” With sharp regression discontinuity, the association estimated is equal to the average causal effect in the population around the threshold. With fuzzy regression discontinuity, where treatment is assigned probabilistically, this measure becomes the intention to treat (ITT), or the effect of eligibility for treatment as determined by the threshold. This is analogous to the ITT commonly estimated in a randomized controlled trial (20–22).

In practice, a bias-variance tradeoff exists in the estimation of these models. While theoretically resulting in the least biased estimate of the causal effect, an analysis restricted only to persons immediately above and below the threshold may have insufficient statistical power. To increase power, information can be borrowed from persons further away from the threshold. However, as the bandwidth around the threshold widens, correct modeling of the functional form of the expected value of the outcome given the assignment variable becomes increasingly important to maintain exchangeability.

Two assumptions must be evaluated and met for estimating the causal effect in the ITT for RDDs. First, the assignment variable must be continuous at the threshold. Second, the so-called continuity assumption (which is that potential outcomes are continuous at the threshold) must hold. When it is met, it implies exchangeability. There can be no other factors at the threshold that would cause a discontinuity in the potential outcomes. If there exist other variables that are discontinuous at the threshold and also affect outcomes, the continuity assumption would be violated and causal inference jeopardized. In practice, when an assignment variable is measured with random noise, we expect this key assumption to be met for persons close to the threshold. Another assumption of RDD, consistency (23) (e.g., that interventions are well-defined) and positivity (24) (e.g., that there are observations in each stratum), is generally expected to be met, since interventions assigned by a threshold rule are generally well-defined, and we expect there to be persons both above and below the threshold (13). One precondition for regression discontinuity is that the threshold rule is known (10). When this precondition is met, the consistency assumption will also hold.

In fuzzy regression discontinuity, an alternative to the ITT estimate is the effect of the exposure itself on outcomes. This measure, called the complier average causal effect (CACE) or local average treatment effect (LATE), can be estimated using instrumental variable approaches (4, 10, 25), where the instrument is treatment assignment by the threshold rule and is used to identify the exogenous variation in actual treatment. The CACE is what would have occurred had everyone in the sample complied with the treatment assignment by the threshold rule. This estimate can be viewed as the “undiluted” effect of treatment, which will only coincide with the ITT under the ideal conditions of perfect compliance (26).

Estimation of the CACE using treatment assignment by the threshold rule as the instrument requires monotonicity—that is, all people who are affected by the instrument are affected by it in the same way. Monotonicity rules out the possibility that for a given change in the instrument, there are both some individuals whose treatment status changes in one direction and other individuals whose treatment status changes in the opposite direction (i.e., there are no so-called defiers). Unfortunately, defiers are not an empirically identifiable population. In general, instrumental variable analyses require the exclusion restriction (4). The exclusion restriction states that the instrument only affects the outcome via treatment status itself. In regression discontinuity, if the continuity assumption has been met, there is no confounding of the threshold (e.g., common causes of the threshold and the outcome) and thus the exclusion restriction will be met (5).

### Inverse probability weighting

Inverse probability weighting of MSMs reduces bias in the presence of time-varying confounding (9). Conventional multiple regression models result in bias in the presence of time-varying confounding, as they rely on conditioning on all covariates in the model. Inverse probability weighting of MSMs overcomes these limitations by creating a pseudopopulation in which there is no association between confounding variables and treatment by estimating the probability of receiving the treatment that the subject actually received at time *k*, conditional on past treatment and risk factor history (9, 27, 28). In the pseudopopulation, under the assumption of no unmeasured confounding (i.e., conditional exchangeability in the original population), the unexposed and exposed are expected to be unconditionally exchangeable, and causal effects can be estimated (2).

The key assumption for identification of causal effects in IPW MSMs is conditional exchangeability. Any unmeasured variables that are associated with both the exposure of interest and the outcome would jeopardize causal inference in this framework. If the exposed and unexposed are conditionally exchangeable in the original population, they will be unconditionally exchangeable in the pseudopopulation, allowing for identification of marginal causal effects. The exposed and unexposed may be conditionally exchangeable if time-varying stabilized inverse probability weights include baseline covariates in the numerator. However, if the assumption of conditional exchangeability (i.e., no unmeasured or residual confounding) does not hold in the original population, the exchangeability assumption will not hold in the pseudopopulation. This assumption is empirically unverifiable. Researchers must rely on subject-matter knowledge to assess the degree to which this assumption is reasonable. The additional identifying assumptions of consistency and positivity must also hold for valid causal inference (23).

### Application to ART initiation and mortality

We applied both the RDD and IPW MSMs to estimate the effect of immediate ART initiation on all-cause mortality in rural South Africa. The ITT for the RDD estimates the effect of immediate ART eligibility on all-cause mortality. The CACE for the RDD estimates the effect of immediate ART initiation in the population of persons who initiated ART because of the threshold rule (e.g., the compliers). The time-invariant MSM estimates the effect of immediate initiation of ART, remaining in care and on ART throughout follow-up. The time-varying MSM estimates the effect of initiating ART during follow-up and remaining on ART and remaining in care such that laboratory values are recorded as compared with never initiating ART during follow-up.

We used data from a large population-based cohort in rural South Africa (29, 30), which is maintained by the Africa Health Research Institute, one of the Wellcome Trust's 5 Africa and Asia programs. The cohort includes data for all patients initiating care at public-sector ART clinics between 2007 and 2011 who presented with a CD4 cell count less than or equal to 350 cells/μL. We excluded patients initiating care after August 2011, as the CD4 cell count threshold changed after this date. Patients with CD4 counts greater than 350 cells/μL at baseline were excluded from all analyses to ensure that the analytical population was the same for all analyses. Data from the public-sector ART clinics were linked to the population-based cohort, in which all members of households in a 438-km^2^ (274-square-mile) area are followed longitudinally. Household response rates exceed 99% (29). Data collected include information on demographic, socioeconomic, and health indicators. Mortality is assessed via verbal autopsy (31). For this analysis, all-cause mortality was the outcome of interest.

ART initiation in this context is assigned via a CD4-count–based threshold rule: Until August 2011, patients with HIV were eligible for ART once their CD4 counts dropped below 200 cells/μL. If patients had a CD4 count above 200 cells/μL, they were deferred from ART until their next monitoring visit, in approximately 6 months. For the regression discontinuity method, we estimated the ITT using a discrete-time hazards model with terms for the gap in CD4 count above and below the threshold. The ITT was estimated for 3 bandwidths of CD4 count around the threshold: ≤350 cells/μL (the entire study sample; *n* = 4,435), 150–250 cells/μL (*n* = 1,304), and 175–225 cells/μL (*n* = 626). We conducted sensitivity analyses to assess whether results were robust to the inclusion of baseline covariates (Web Appendix 2). To estimate the effect of initiation of ART itself in the RDD, we calculated the CACE in each of the 3 bandwidths described above (10, 13). To estimate the CACE, we used a discrete-time hazards model using *ivprobit* in Stata (StataCorp LLC, College Station, Texas). Patients were followed from the date of their first CD4 count in the HIV care system (as a proxy for the date of initiation of HIV care) to the date of their last observance in the surveillance system, classified as either the end of follow-up (censored) or death.

Using the IPW MSM, we estimated the effect of initiating treatment at entry into care (within a 3-month grace period) versus not. Participants were followed until the end of follow-up (December 2014) or death, whichever came first. We also estimated the effect of time-updated ART status. Because not all participants had laboratory measurements taken every 6 months per monitoring guidelines and some were lost to care entirely, in the time-varying analysis we censored persons who had not had a laboratory measurement for 12 months or more. An additional model included both censoring at 12 months and an interaction term for ART status × time, which allowed for estimation of heterogeneity of effects over time. Time-invariant models were not censored.

For the model estimating the effect of ART initiation at entry into care, we first estimated inverse probability weights with the following baseline covariates: age, sex, CD4 count, educational status, household wealth (estimated as the first principal component in a principal components analysis of 32 household assets and characteristics and discretized into quintiles (29)), distance from the patient’s place of residence to the clinic (in kilometers), and place of residence (whether the patient lived in an urban or rural area). Although we permitted a 3-month grace period to allow for minor delays in initiating ART, only baseline CD4 cell count was used in the calculation of the inverse probability weights, as CD4 count monitoring is typically done at 6-month intervals. A discrete-time hazards model was then used to fit the MSM, without adjustment for any covariates. The discrete-time hazards model was utilized to accommodate time-varying weights for the time-varying MSM, and an identical approach was used for all other models to maintain consistency.

To compare the regression discontinuity model with models of the effect of time-varying ART status on mortality, we conducted a time-varying analysis with inverse probability weighting. Methods used for these models are included in Web Appendix 3. Due to the noncollapsibility of the hazard ratio, results of the time-varying analysis with stabilized weights are not directly comparable to results derived from the time-invariant MSM. An additional analysis included accounting for time-varying confounding and informative censoring using the joint distribution of inverse-probability-of-treatment and inverse-probability-of-censoring weights (Web Appendix 4).

## RESULTS

**Figure 1.**
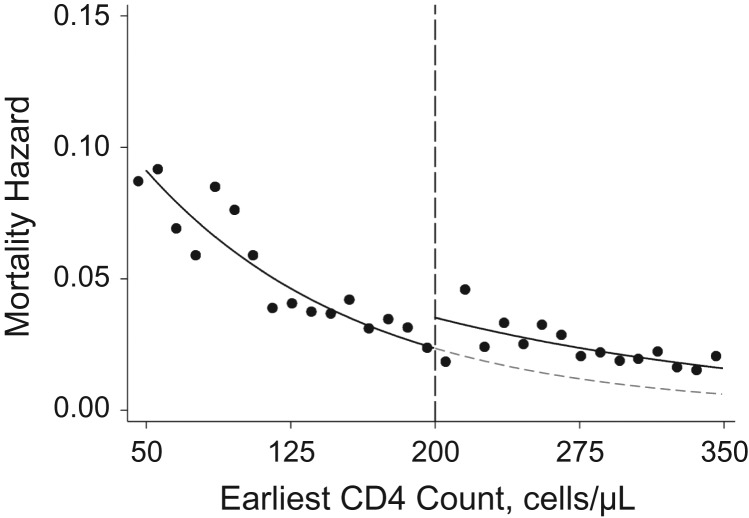
Hazard of mortality among persons with human immunodeficiency virus infection according to baseline CD4 cell count, KwaZulu-Natal, South Africa, 2007–2011. Black dots (●) indicate the raw mortality hazard (incidence) for each 10-cell/μL group. The solid lines are fitted regression lines showing the incidence of mortality as a function of the earliest CD4 cell counts above and below the threshold (dashed line). The dashed gray line is the projection for the curve below the threshold, which is the estimate of what mortality incidence would have been for persons who were above the threshold (and thus not eligible for immediate initiation of antiretroviral therapy (ART)) if they had actually been eligible for ART immediately. The discontinuity at the threshold is the estimate of the effect of ART eligibility on mortality incidence.

A total of 4,435 persons initiated care between 2007 and 2011 with a baseline CD4 count less than or equal to 350 cells/μL. Of these, 935 participants died over the course of 19,269 person-years of follow-up (mortality incidence rate = 4.9 per 100 person-years). Table 2 and Web Table 1 show the distribution of baseline covariates for persons above and below the threshold. Of the 935 deaths, 734 were among persons who were eligible for ART at baseline (incidence rate = 6.6 per 100 person-years) and 201 occurred among those who were not eligible (2.5 per 100 person-years). Mortality decreased as baseline CD4 count increased, and there was evidence of a discontinuity at the 200-cells/μL threshold (Figure 1).

**Table 2.** Baseline Characteristics of Participants in a Study of Immediate Initiation of Antiretroviral Therapy Among Persons With Human Immunodeficiency Virus Infection (*n* = 4,435), KwaZulu-Natal, South Africa, 2007–2011

Characteristic	Eligibility for ART Initiation at Baseline					
	Below Threshold (Eligible) (*n* = 2,751)			Above Threshold (Ineligible) (*n* = 1,684)		
	No. of Persons	%	Median (IQR)	No. of Persons	%	Median (IQR)
Age, years			33.1 (27.5–41.4)			31.3 (24.8–40.3)
Female sex	1,790	65.1		1,278	75.9	
Baseline CD4 cell count, cells/μL			101 (49–140)			268 (233–306)
Asset index quintile^a^						
Lowest	594	21.6		402	23.9	
Second lowest	571	20.8		357	21.2	
Middle	488	17.7		315	18.7	
Second highest	404	14.7		239	14.2	
Highest	347	12.6		241	14.3	
Missing data	347	12.6		130	7.7	
Distance to nearest clinic, km			2.6 (1.5–3.4)			2.4 (1.5–3.5)
Place of residence						
Rural	1,249	45.4		813	48.3	
Periurban	918	33.4		553	32.8	
Urban	252	9.2		189	11.2	
Missing data	332	12.1		129	7.7	
Educational attainment, years						
≤7 (none or primary)	920	33.4		564	33.5	
8–12 (secondary)	1,523	55.4		951	56.5	
>12 (tertiary)	246	8.9		133	7.9	
Missing data	62	2.3		36	2.1	

Abbreviations: ART, antiretroviral therapy; IQR, interquartile range.

^a^ Household wealth was estimated as quintiles of the first components identified by principal components analysis of 32 household assets and characteristics.

**Figure 2.**
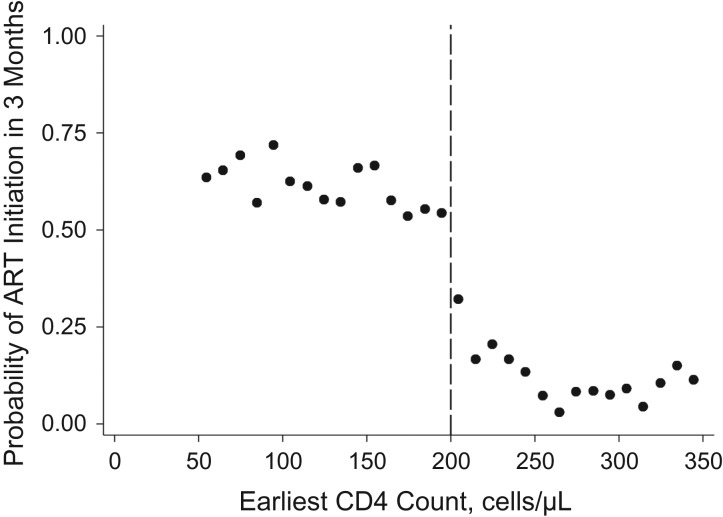
Probability of antiretroviral therapy (ART) initiation within 6 months of entering care among persons with human immunodeficiency virus infection, according to baseline CD4 cell count, KwaZulu-Natal, South Africa, 2007–2011. The probability of ART initiation within 6 months of the earliest CD4 cell count was calculated by baseline CD4 cell count as the number of persons in a given 10-cell/μL group over the total number of persons in that group. A discontinuity in the probability of ART initiation is evident at the 200-cells/μL threshold.

Figure 2 demonstrates evidence of discontinuity in the probability of initiating ART within 6 months by baseline CD4 count. Baseline characteristics for the overall sample were roughly balanced between persons who were eligible for treatment at baseline and those who were not eligible (Table 2) but were more closely balanced for persons closer to the threshold (Web Table 1). Sensitivity analyses were conducted with additional functional forms for CD4 counts above and below the threshold, including 1) a squared functional form for CD4 counts below the threshold; 2) a restricted cubic spline at 125 cells/μL (75 cells/μL below the threshold); 3) the addition of age at baseline and sex as covariates in the model (Web Table 2).

**Figure 3.**
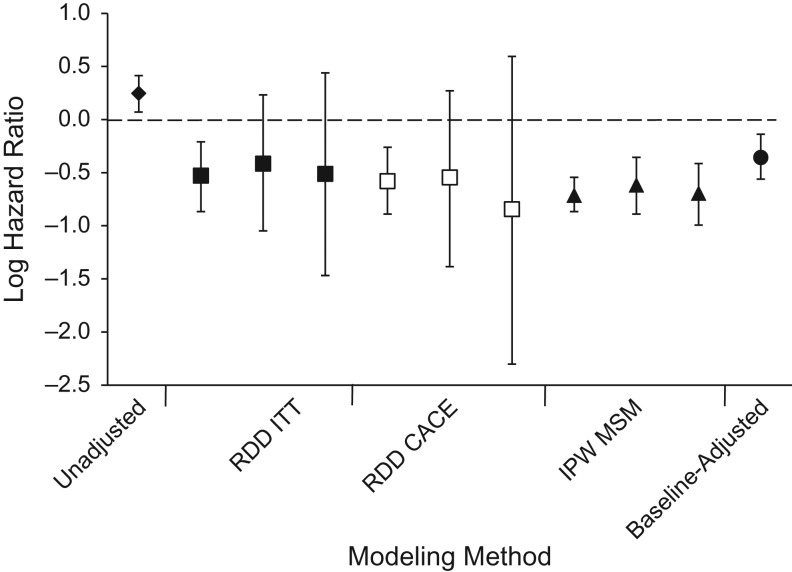
Primary analysis results for the relationship between antiretroviral therapy (ART) and mortality among persons with human immunodeficiency virus infection, KwaZulu-Natal, South Africa, 2007–2011. Results were derived using the regression discontinuity design (RDD) for both the intention-to-treat (ITT) effect (■) and the complier average causal effect (CACE; □) at the ≤350-cells/μL, 50- to 350-cells/μL, and 150- to 250-cells/μL bandwidths (left to right); inverse-probability–weighted (IPW) marginal structural models (MSMs) (from left to right, baseline time-invariant ART initiation, time-updated ART status censoring persons without laboratory tests at 12 months, and time-updated ART status censoring persons without laboratory tests at 12 months with inverse-probability-of-censoring weights applied) (▲); the unadjusted effect of ART on mortality (♦); and the effect adjusted for baseline covariates (●). Bars, 95% confidence intervals.

Figure 3 graphically displays the results of each analysis. In the regression discontinuity model at a bandwidth of ≤350 cells/μL, there was a 41% reduction in mortality with immediate eligibility for ART (hazard ratio (HR) = 0.59, 95% confidence interval (CI): 0.42, 0.81) (Figure 2, Table 3), which was lowered to a 30% reduction in a model with a restricted cubic spline (HR = 0.70, 95% CI: 0.46, 1.05) and in a model with a squared functional form for CD4 count (HR = 0.70, 95% CI: 0.43, 1.13). These results were similar in magnitude to the effect estimates at a narrower bandwidth of 150–250 cells/μL (HR = 0.66, 95% CI: 0.35, 1.26). The CACE demonstrated a 44% reduction in mortality with immediate initiation of ART among the compliers at the widest bandwidth (HR = 0.56, 95% CI: 0.41, 0.77). These estimates were robust to the inclusion of baseline covariates in the model (Web Table 2).

**Table 3.** Primary Analysis Results for Regression Discontinuity and Inverse-Probability–Weighted Analyses of Immediate Initiation of Antiretroviral Therapy Among Persons With Human Immunodeficiency Virus Infection, KwaZulu-Natal, South Africa, 2007–2011

Model and CD4 Cell Count	HR	95% CI
Unadjusted	1.28	1.08, 1.51
Regression discontinuity—ITT		
≤350 cells/μL	0.59	0.42, 0.81
150–250 cells/μL	0.66	0.35, 1.26
175–225 cells/μL	0.50	0.23, 1.55
Regression discontinuity—CACE		
≤350 cells/μL	0.56	0.41, 0.77
150–250 cells/μL	0.58	0.25, 1.31
175–225 cells/μL	0.43	0.10, 1.81
Inverse probability weighting		
Time-invariant ART status	0.49	0.42, 0.58
Time-varying ART status^a^	0.54	0.41, 0.70
Time-varying ART status with censoring weights^b^	0.50	0.37, 0.66

Abbreviations: ART, antiretroviral therapy; CACE, complier average causal effect; CI, confidence interval; HR, hazard ratio; ITT, intention to treat.

^a^ Censored at a 12-month gap in laboratory values.

^b^ Censored at a 12-month gap in laboratory values and accounting for censoring with inverse-probability-of-censoring weights applied.

In the IPW MSM, initiating ART within a 3-month grace period from study entry was associated with a 51% reduction in all-cause mortality (HR = 0.49, 95% CI: 0.42, 0.58) and a 46% reduction in a time-varying model censoring persons without a laboratory test in the previous 12 months (HR = 0.54, 95% CI: 0.41, 0.70). In the censored model with an ART status × time-since-initiation interaction term, there was significant evidence of an increase in the protective effect of ART over time (per year, HR = 0.84, 95% CI: 0.76, 0.94).

## DISCUSSION

The RDD and the IPW MSM yielded consistent results, indicating an approximately 40%–50% reduction in mortality due to immediate initiation of ART. These results provide both consistent and complementary information. The CACE and the time-invariant MSM estimate the effect of immediate ART initiation (within 3 months of entry into care) on all-cause mortality. The CACE found a 44% reduction in all-cause mortality, as compared with 51% with the time-invariant MSM, with completely overlapping confidence intervals. The time-varying MSM, a commonly used method for modeling ART status as an exposure due to the potential for time-varying confounding, was similarly consistent with the previous 2 approaches. Prior studies have established the efficacy of ART for prevention of mortality, and CD4 count is a well-known confounder of this relationship. We chose the causal effect of ART on mortality for our study using multiple causal inference methods, because this relationship is well-understood and thus provides a good example with which to demonstrate the usefulness of a methodological approach. In future applications, triangulation of causal inference across multiple methods may be most valuable when the preexisting evidence is weak or inconsistent. In these cases, inconsistent findings across different causal methods will be more likely than in our application, and consistent findings thus have greater potential to substantially strengthen the evidence on causality.

While the broad causal question of interest in the present application was the relationship between ART and mortality, each study design presented herein answered a subtly different specific causal question. Each of these results has a slightly different interpretation. The unadjusted association showed an increased risk of mortality with ART use, which is expected given the strong confounding by CD4 count. Each method of causal inference evaluated here showed that, as expected, ART use was protective against mortality. Taken together, these results demonstrate the robustness of the causal effect of ART on mortality in “real life.” Using a large population-based cohort without the resources of a carefully conducted randomized controlled trial, we demonstrated a large decrease in mortality with the use of ART. The use of multiple approaches for causal inference also strengthens the evidence arising from this study in comparison with most cohort studies, which do not use such approaches.

The different methods we used generate different types of causal effect size estimates. Time-invariant inverse probability weighting of MSMs determines the estimate in the entire study population and thus can be generalized across the entire distribution of CD4 cell counts. Notably, in the time-varying model, the estimate is conditional on baseline covariates. In the RDD, generalizability will depend on additional assumptions. In the presence of heterogeneity of treatment effects according to the assignment variable (in this application, CD4 count), the RDD effect may have limited generalizability. In the RDD literature, 3 perspectives on the generalizability of effects have been posited (5, 12). First is that the effect size is generalizable to the entire study population and is an average treatment effect (32). The assumption required for this interpretation is that the functional form of the potential outcomes (e.g., had everyone been treated versus had no one been treated) are known. However, this is a strong and untestable assumption. The second perspective is that the effect estimated under the RDD is only generalizable locally, within an arbitrary region around the threshold. The third perspective is that the effect size estimated under the RDD represents a weighted average of the treatment effect in the entire study population, with weights that are proportional to the probability of an individual’s value of the assignment variable (CD4 count) being in the neighborhood of the threshold (33). Given the strong assumptions required for the first perspective, the IPW estimate, which is an average treatment effect, could differ from the regression discontinuity estimate if the relationship between ART and mortality is stronger at lower CD4 counts (i.e., in persons who are sicker at baseline), compared with those whose baseline CD4 count is 200 cells/μL.

For both methodological approaches, the data we used for these analyses had significant strengths. The public-sector ART clinic in this region (the Hlabisa HIV Treatment and Care Programme) provides the vast majority of HIV care in the area, and thus misclassification of exposure is likely to have been minimal. Follow-up information was very complete in our study, because it was collected through one of Africa’s largest and most rigorous population-based cohorts. The cohort covers the entire population in the catchment areas of the clinics through which patients were recruited for our study, and data on mortality have been collected on an ongoing basis for over 15 years. Death registration in the cohort is near-complete (34). As a result, we still observed our outcome of interest in nearly all of the patients who were lost to follow-up from clinical HIV care. Use of complete, high-quality data allows for direct comparison of estimates generated by the two approaches, specifically in how they each meet the exchangeability requirement for causal inference from observational data, as well as the generalizability of each estimate.

The effect estimates from the RDD and MSM were not identical, but they were similar in magnitude, providing strong evidence of a protective effect of ART against all-cause mortality among HIV-infected persons receiving care in rural South Africa. The fact that the IPW MSM estimate was similar to the RDD estimate suggests that the regression discontinuity effect may be generalizable to a wider range of CD4 counts from the 200-cells/μL threshold. When covariate data are available and well-measured, inclusion of an IPW MSM as a sensitivity analysis may yield greater information than the regression discontinuity effect alone. The use of both models provides estimation of effects with alternative exchangeability assumptions and yields complementary information regarding external validity. Although the exact specification of the causal question may differ slightly, using multiple methods to address the same overarching question adds additional robustness to the interpretation of results. When the data allow, approaches that have different assumptions for validity should be used routinely to strengthen confidence in causal effect estimates.

## Supplementary Material

Web Material

## Abbreviations

ART: antiretroviral therapy
CACE: complier average causal effect
CD4: cluster of differentiation 4
CI: confidence interval
HIV: human immunodeficiency virus
HR: hazard ratio
IPW: inverse-probability–weighted
ITT: intention to treat
MSM: marginal structural model
RDD: regression discontinuity design

## References

[1] Hernán MA, Robins JM Estimating causal effects from epidemiological data. J Epidemiol Community Health. 2006;60(7):578–586. 16790829 10.1136/jech.2004.029496 PMC2652882

[2] Hernan MA, Robbins JM Causal inference book. 2015 https://www.hsph.harvard.edu/miguel-hernan/causal-inference-book/. Accessed September 18, 2015.

[3] Rockers PC, Røttingen JA, Shemilt I, et al Inclusion of quasi-experimental studies in systematic reviews of health systems research. Health Policy. 2015;119(4):511–521. 25776033 10.1016/j.healthpol.2014.10.006

[4] Hernán MA, Robins JM Instruments for causal inference: an epidemiologist’s dream? Epidemiology. 2006;17(4):360–372. 16755261 10.1097/01.ede.0000222409.00878.37

[5] Bärnighausen T, Oldenburg C, Tugwell P, et al Quasi-experimental study designs series—paper 7: assessing the assumptions. J Clin Epidemiol. 2017;89:53–66. 28365306 10.1016/j.jclinepi.2017.02.017

[6] Severe P, Juste MA, Ambroise A, et al Early versus standard antiretroviral therapy for HIV-infected adults in Haiti. N Engl J Med. 2010;363(3):257–265. 20647201 10.1056/NEJMoa0910370 PMC3676927

[7] Grinsztejn B, Hosseinipour MC, Ribaudo HJ, et al Effects of early versus delayed initiation of antiretroviral treatment on clinical outcomes of HIV-1 infection: results from the phase 3 HPTN 052 randomised controlled trial. Lancet Infect Dis. 2014;14(4):281–290. 24602844 10.1016/S1473-3099(13)70692-3 PMC4144040

[8] INSIGHT START Study Group, Lundgren JD, Babiker AG, et al Initiation of antiretroviral therapy in early asymptomatic HIV infection. N Engl J Med. 2015;373(9):795–807. 26192873 10.1056/NEJMoa1506816 PMC4569751

[9] Hernán MA, Brumback B, Robins JM Marginal structural models to estimate the causal effect of zidovudine on the survival of HIV-positive men. Epidemiology. 2000;11(5):561–570. 10955409 10.1097/00001648-200009000-00012

[10] Bor J, Moscoe E, Mutevedzi P, et al Regression discontinuity designs in epidemiology: causal inference without randomized trials. Epidemiology. 2014;25(5):729–737. 25061922 10.1097/EDE.0000000000000138 PMC4162343

[11] Moscoe E, Bor J, Bärnighausen T Regression discontinuity designs are underutilized in medicine, epidemiology, and public health: a review of current and best practice. J Clin Epidemiol. 2015;68(2):122–133. 25579639 10.1016/j.jclinepi.2014.06.021

[12] Bor J, Moscoe E, Bärnighausen T Three approaches to causal inference in regression discontinuity designs. Epidemiology. 2015;26(2):e28–e30. 10.1097/EDE.0000000000000256 25643120

[13] Oldenburg CE, Moscoe E, Bärnighausen T Regression discontinuity for causal effect estimation in epidemiology. Curr Epidemiol Rep. 2016;3:233–241. 27547695 10.1007/s40471-016-0080-x PMC4978750

[14] Vandenbroucke JP, le Cessie S Commentary: regression discontinuity design: let’s give it a try to evaluate medical and public health interventions. Epidemiology. 2014;25(5):738–741. 25076150 10.1097/EDE.0000000000000145

[15] Thistlethwaite DL, Campbell DT Regression-discontinuity analysis: an alternative to the ex post factor experiment. J Educ Psychol. 1960;51(6):309–317.

[16] Almond D, Doyle JJ, Kowalski AE, et al Estimating marginal returns to medical care: evidence from at-risk newborns. Q J Econ. 2010;125(2):591–634. 20634927 10.1162/qjec.2010.125.2.591 PMC2903901

[17] Smith LM, Strumpf EC, Kaufman JS, et al The early benefits of human papillomavirus vaccination on cervical dysplasia and anogenital warts. Pediatrics. 2015;135(5):e1131–e1140. 25917991 10.1542/peds.2014-2961

[18] Smith LM, Kaufman JS, Strumpf EC, et al Effect of human papillomavirus (HPV) vaccination on clinical indicators of sexual behaviour among adolescent girls: the Ontario Grade 8 HPV Vaccine Cohort Study. CMAJ. 2015;187(2):E74–E81. 25487660 10.1503/cmaj.140900 PMC4312170

[19] Shoag J, Halpern J, Eisner B, et al Efficacy of prostate-specific antigen screening: use of the regression discontinuity in the PLCO Cancer Screening Trial. JAMA Oncol. 2015;1(7):984–986. 26291583 10.1001/jamaoncol.2015.2993

[20] Murnane PM, Brown ER, Donnell D, et al Estimating efficacy in a randomized trial with product nonadherence: application of multiple methods to a trial of preexposure prophylaxis for HIV prevention. Am J Epidemiol. 2015;182(10):848–856. 26487343 10.1093/aje/kwv202 PMC4634306

[21] Hernán MA, Hernández-Díaz S Beyond the intention-to-treat in comparative effectiveness research. Clin Trials. 2012;9(1):48–55. 21948059 10.1177/1740774511420743 PMC3731071

[22] Shrier I, Steele RJ, Verhagen E, et al Beyond intention to treat: what is the right question? Clin Trials. 2014;11(1):28–37. 24096636 10.1177/1740774513504151

[23] Hernán MA, Taubman SL Does obesity shorten life? The importance of well-defined interventions to answer causal questions. Int J Obes (Lond). 2008;32(suppl 3):S8–S14. 10.1038/ijo.2008.82 18695657

[24] Westreich D, Cole SR Invited commentary: positivity in practice. Am J Epidemiol. 2010;171(6):674–677. 20139125 10.1093/aje/kwp436 PMC2877454

[25] Tchetgen Tchetgen EJ, Walter S, Vansteelandt S, et al Instrumental variable estimation in a survival context. Epidemiology. 2015;26(3):402–410. 25692223 10.1097/EDE.0000000000000262 PMC4387894

[26] Imbens GW, Angrist JD Identification and estimation of local average treatment effects. Econometrica 1994;62(2):467–475.

[27] Cole SR, Hernán MA Constructing inverse probability weights for marginal structural models. Am J Epidemiol. 2008;168(6):656–664. 18682488 10.1093/aje/kwn164 PMC2732954

[28] Cole SR, Hernán MA, Margolick JB, et al Marginal structural models for estimating the effect of highly active antiretroviral therapy initiation on CD4 cell count. Am J Epidemiol. 2005;162(5):471–478. 16076835 10.1093/aje/kwi216

[29] Tanser F, Hosegood V, Bärnighausen T, et al Cohort profile: Africa Centre Demographic Information System (ACDIS) and population-based HIV survey. Int J Epidemiol. 2008;37(5):956–962. 17998242 10.1093/ije/dym211 PMC2557060

[30] Houlihan CF, Bland RM, Mutevedzi PC, et al Cohort profile: Hlabisa HIV Treatment and Care Programme. Int J Epidemiol. 2011;40(2):318–326. 20154009 10.1093/ije/dyp402 PMC3195268

[31] Bor J, Rosen S, Chimbindi N, et al Mass HIV treatment and sex disparities in life expectancy: demographic surveillance in rural South Africa. PLoS Med. 2015;12(11):e1001905. 26599699 10.1371/journal.pmed.1001905 PMC4658174

[32] Rubin DB Assignment to treatment group on the basis of a covariate. J Educ Stat. 1977;2(1):1–26.

[33] Lee DS, Lemieux T Regression discontinuity designs in economics. J Econ Lit. 2010;48(2):281–355.

[34] Bor J, Herbst AJ, Newell ML, et al Increases in adult life expectancy in rural South Africa: valuing the scale-up of HIV treatment. Science. 2013;339(6122):961–965. 23430655 10.1126/science.1230413 PMC3860268

