# Supplementary material for: Antiretroviral Therapy and Mortality in Rural South Africa: A Comparison of Causal Modeling Approaches
Source: Am J Epidemiol. 2018 Mar 23;187(8):1772–9. doi: 10.1093/aje/kwy065 (PMC6070080; doi:10.1093/aje/kwy065)
Supplement: Web Material [file kwy065_oldenburg_web_material_final.pdf]

WEB MATERIAL

In this material, we present results of sensitivity analyses for the regression discontinuity and marginal structural model analyses. The purpose of these analyses and additional information is to assess the plausibility of assumptions required for causal inference.

Web Appendix 1

Web Table 1 displays the distribution of baseline covariates for individuals in the narrowest bandwidth around the threshold, 150 to 250 cells/μl. Overall, the covariates were well balanced above and below the threshold. Of note, although there was an approximately 10% difference in the proportion of individuals who were female below versus above the threshold in the overall sample, approximately 70% of individuals above and below the threshold were female for individuals closer to the threshold. These results indicate that the data generating process is operating as expected. If, at the area around the threshold, the threshold is truly a source of exogenous variation in treatment assignment, we expect the distribution of covariates to be roughly balanced above and below the threshold. Importantly, that the balance of covariates appears to be closer to random for individuals presenting to care at a narrower bandwidth around the threshold underscores the importance of interpretation of effects from the regression discontinuity model as local effects. Substantial, and untestable, assumptions are required to interpret the effects recovered from the regression discontinuity model as global effects.

Web Table 1. Baseline characteristics for the study sample at 150–250 cells/μl bandwidth (*n* = 1,304), KwaZulu-Natal, South Africa, 2007–2011

	**Below Threshold (Eligible) (*n* = 676)**	**Above Threshold (Ineligible) (*n* = 628)**
Median age, years (IQR)	32 (27–41)	34 (27–43.5)
Female sex	483 (71.6%)	464 (73.9%)
Median baseline CD4 cell count, cells/μL (IQR)	173 (162–186)	225 (213–236.5)
Asset index quintile		
Lowest	137 (20.3%)	149 (23.7%)
Second lowest	141 (20.9%)	141 (22.5%)
Middle	127 (18.8%)	109 (17.4%)
Second highest	113 (16.7%)	90 (14.3%)
Highest	88 (13.0%)	94 (15.0%)
Missing	70 (10.4%)	45 (7.2%)
Distance to nearest clinic, km (IQR)	2.2 (1.3–3.4)	2.2 (1.4–3.5)
Residence		
Rural	291 (43.1%)	215 (34.2%)
Peri-Urban	254 (37.6%)	299 (47.6%)
Urban	63 (9.3%)	70 (11.2%)
Missing	68 (10.1%)	44 (7.0%)
Educational attainment		
None or primary (0-7 years)	216 (32.0%)	210 (33.4%)
Secondary (8-2 years)	382 (56.5%)	359 (57.2%)
Tertiary	60 (8.9%)	46 (7.3%)
Missing	18 (2.7%)	13 (2.1%)

Web Appendix 2

In this sensitivity analysis, we included baseline covariates in the intention-to-treat (ITT) regression discontinuity model for the effect of ART on mortality. This sensitivity analysis tests the assumption that the distribution of baseline covariates is balanced above and below the threshold, and that the effect of ART on mortality is not being driven by a confounding variable. If the estimates are not being driven by confounding by an imbalance of other covariates, we expect the point estimate will not change compared to the model without the inclusion of baseline covariates.

In the sensitivity analysis presented in Web Table 2, we report age- and sex-adjusted ITT as well as the age- and sex-adjusted ITT that includes a restricted cubic spline for CD4 count. These models show very little movement in the point estimate. The change in hazard ratio for most bandwidths was 0.01, and the largest change was 0.02. These results indicate that the results of the regression discontinuity analysis were robust to the inclusion of two of the strongest determinants of mortality, age and sex, suggesting that the effect at the threshold was not driven by endogenous factors. Although this sensitivity analysis cannot rule out the influence of unmeasured factors, it gives strong support to the data generating process behaving as expected. Close to the threshold, the variation in treatment assignment appears to be primarily driven by chance, rather than by other causal processes.

Web Table 2. Intention-to-treat estimates (and 95% confidence intervals) adjusted for baseline covariates, KwaZulu-Natal, South Africa, 2007–2011

Bandwidth	Baseline Covariate-Adjusted ITT	Baseline Covariate-Adjusted ITT with Restricted Cubic Spline
0–350	0.58 (0.42 to 0.81)	0.72 (0.48 to 1.08)
50–350	0.64 (0.45 to 0.91)	0.69 (0.44 to 1.07)
150–250	0.69 (0.36 to 1.30)	

Web Appendix 3

For the time-varying analysis, the parameters of a marginal structural model were estimated by first estimating stabilized inverse probability weights, with the same baseline covariates as the time-invariant model and time-updated CD4 count, household wealth, distance to clinic, and urban or rural residence. The weights were truncated at a maximum value of 10 to avoid influence of outliers on the variance. Less than 0.1% of weights were truncated, and the untruncated weights had a mean of 1.01 (range, 0.02 to 207). After truncation, the distribution of stabilized inverse probability of treatments weights had a mean of 1.003 (1st percentile: 0.85, 99th percentile: 1.13). A discrete time hazards model was used to estimate the marginal structural model. Because the numerator of the stabilized inverse probability of treatment weights estimated the probability of treatment at time t as a function of baseline covariates, the time-varying marginal structural model was fit including baseline covariates.

Web Appendix 4

This sensitivity analysis includes weights for both selection bias (censoring) and confounding in the model in which individuals are censored if they have not had a laboratory test after 12 months. In this model, the joint distribution of weights for censoring and confounding is estimated by first estimating stabilized inverse probability weights for confounding as described in the main text. We then repeat the procedure to estimate weights predicting the probability of remaining uncensored. This joint distribution of weights creates a pseudopopulation in which everyone remains uncensored and there is no association between time-varying confounding variables and treatment. Under the assumption of no unmeasured confounding, the effect can be interpreted as the effect of ART on mortality had everyone been uncensored, or had everyone in the population received ART, compared to if no one had received ART.

In the inverse probability-weighted model adjusted for both confounding and censoring, had everyone received ART, there would have been 50% of the hazards of mortality had no one received ART (HR = 0.50, 95% CI: 0.37, 0.66). This was similar in magnitude to the model in which individuals were censored if they had not had a laboratory measurement for 12 months but there was no adjustment for censoring (HR = 0.54, 95% CI: 0.41, 0.70). Both of these models indicated a stronger effect of ART than the model in which there was no censoring (HR = 0.62, 95% CI: 0.50, 0.76). This is as expected, as in the uncensored model, CD4 counts, a major cause of time-varying confounding of the association between ART status and mortality, are carried forward. This can result in residual confounding, which may have biased effects towards the null in this case.
